# Two cell cycle blocks caused by iron chelation of neuroblastoma cells: separating cell cycle events associated with each block

**DOI:** 10.1002/phy2.176

**Published:** 2013-12-06

**Authors:** Gamini Siriwardana, Paul A. Seligman

**Affiliations:** 1 Division of Hematology, Hematologic Malignancies and Stem Cell Transplantation, University of Colorado School of Medicine, 12700 E. 19th Avenue, Room 9122RC 2, MS B170, Aurora, 80045, Colorado

**Keywords:** Cell cycle, iron chelation, neuroblastoma

## Abstract

Studies have presented evidence that besides the well described S phase block, treatment of cancer cell lines with the iron chelator deferrioxamine (DFO) also results in an earlier block in G_1_ phase. In this article, measurements of cell cycle regulatory proteins define this block at a very specific point in G_1_. DFO treatment results in markedly decreased cyclin A protein levels. Cyclin E levels that accumulate in early to mid‐G_1_ are increased in cells treated with DFO as compared to the resting cells. The DFO S phase block is shown after cells are arrested at G_1_/S by (aphidicolin) then released into DFO. The same S phase block occurs with DFO treatment of a neuroblastoma cell line relatively resistant to the G_1_ DFO block. These experiments clearly differentiate the S phase DFO block from the earlier block pinpointed to a point in mid‐G_1_, before G_1_/S when cyclin E protein increases but before increased cyclin A synthesis. Apoptosis was observed in cells inhibited by DFO at both cell cycle arrest points.

## Abstract (precis)

Iron chelation inhibits cell proliferation at two phases in the cell cycle. The block in G1 phase is now separated from the block in S phase by specific differences in expression of cell cycle regulatory events.

## Introduction

Iron is required for cellular proliferation (Robbins and Pederson 1970; Rudland et al. 1977). Cancer cells, particularly rapidly growing cells, are especially sensitive to iron depletion (Kontoghiorghes et al. 1986; Nghia and Richardson 2002). Measurements of iron status in epidemiologic studies have shown a lower incidence of cancer in iron deplete individuals (Zacharski et al. 2008), and a higher incidence in iron overload (Lenarduzzi et al. 2013). For example, gallium infusion has been utilized as a chemotherapeutic agent and its efficacy is directly related to iron depletion of cancer cells (Seligman and Crawford 1991; Seligman et al. 1992).

Numerous studies have shown that iron chelation results in inhibition of cellular proliferation. The agent used as the “gold standard” as a chelator for many years is deferrioxamine (DFO) (Lederman et al. 1984; Blatt and Stitely 1987). Neuroblastoma cell lines taken from patients with this childhood tumor that is relatively resistant to chemotherapy compared to other childhood tumors are especially sensitive to growth inhibition by iron depletion (Blatt et al. 1988; Helson and Helson 1992; Brodie et al. 1993). In the past, DFO has been used clinically as a treatment for patients with neuroblastoma (Donfrancesco et al. 1992; Philip 1992). New chelators, with more potential as chemotherapeutic agents, have improved iron chelation efficacy but also have lipophilicity (Yu et al. 2012; Lui et al. 2013), a characteristic that may confound some biochemical processes *in vitro*.

Neuroblastoma cell lines, therefore, are ideal for measuring the specific mechanisms associated with inhibition of cellular proliferation caused by iron depletion, including the use of DFO. A well‐known mechanism by which iron depletion causes inhibition of cellular proliferation is inhibition of the iron requiring enzyme, ribonucleotide reductase (RR). RR is necessary for DNA synthesis, and when inhibited, cells arrest in S phase of the cell cycle (Eriksson et al. 1984; Hoyes et al. 1992). A number of studies have also shown that certain normal and cancer cell lines, especially those sensitive to iron depletion, exhibit a cell cycle arrest point in G_1_ (or G_1_/S phase) (Brodie et al. 1993; Nghia and Richardson 2002). Numerous mechanisms have been advanced to elucidate how iron depletion results in cell cycle arrest. Moreover, it is also known that there are many cellular functions that require iron; some are associated with cellular proliferation but many are not. It is not surprising, therefore, that there may be more than one mechanism. It has been difficult, however, to distinctly separate mechanisms associated with this S phase block from those mechanisms that are associated with the G_1_ block.

In the current studies, we used sequential blocking by aphidicolin, hydroxyurea, and DFO to better define the two cell cycle blocks caused by DFO. Aphdicolin inhibits cell cycle at G/S by inhibiting the activity of DNA polymerase (Krokan et al. 1981). Hydroxyurea blocks the cell cycle at G_1_/S (or early S) by inhibiting the activity of RR by quenching the free radicals of its M2 subunit (Tyrsted 1982; Yarbro 1992; Koç et al. 2004). Blocking the cells sequentially with the above compounds, we are able to better separate the two DFO arrest points by measuring cyclins that regulate activation (phosphorylation) of the cyclin‐dependent kinases that allow for progression through the cell cycle. Using a neuroblastoma cell line that is relatively resistant to the G_1_ inhibitory effects of DFO (similar to a glioblastoma cell line we have previously described (Brodie et al. 1993), as well as cells arrested at G_1_/S phase by aphidicolin, we are able to separate the mechanisms associated with the G_1_ DFO block compared to the S phase DFO block.

## Materials and Methods

DFO, hydroxyurea, and aphidicolin were obtained from CIBA‐GEIGY Canada. The DFO stock solution was reconstituted in distilled water containing 100 mm DFO. DFO treatments were usually given at 100 *μ*mol/L dose. We have previously shown that 10 *μ*m DFO causes significant growth inhibition and G_1_ arrest of neuroblastoma, (Brodie et al. 1993) but this higher dose was given because earlier time points were used in many experiments and differences in iron contamination of the media could be accounted for. Hydroxyurea was made up to a 100 mmol/L solution in sterile phosphate buffered saline (PBS). Aphidicolin was prepared as a 2.5 mg/mL stock solution in dimethyl sulfoxide (DMSO). All aphidicolin treatments were given at a 2.5 *μ*g/L dose. Antibodies for cyclin A, cyclin E, cyclin D1, and RB along with beta‐actin were obtained from Santa Cruz Biotechnology (Santa Cruz, CA).

SKNSH and SKNAS (ATCC HTB‐11) human neuroblastoma cell lines were maintained in stock culture in Roswell Park Memorial Institute (RPMI) 1640 and 10% fetal calf serum (FCS). This medium is used as control medium (CM). Once the cells reached a state of confluency (contact inhibition) the cells were kept in RPMI 1640 with no serum for 20–24 h. When DFO was preincubated with the cells before subculture the DFO was added to the serum‐free medium. Changes in cell growth after subculture and under various tissue culture conditions were assessed by cell counts using a cytometer (Brodie et al. 1993). Cell cycle analysis was performed by staining with propidium iodide and analyzed by flow cytometry as previously described (Brodie et al. 1993).

For immunoblot analysis, cells were grown in 35‐mm tissue culture dishes in 3 mL of RPMI 1640/10% FCS. Harvesting of cells after various treatments was done by aspirating the growth medium, followed by washing of the cells with cold PBS and adding 200 *μ*L of SDS sample buffer. Proteins were resolved by subjecting 40 *μ*L samples to sodiumdodecyl sulfate polyacrylamide gel electrophoresis (SDS‐PAGE) (Tyrsted 1982). Proteins were transferred to (EMD Millipore, Billerica, MA) polyvinylidene difluoride (PVDF) membranes in a 192 mmol/L lysine, 25 mmol/L Tris and 20% methanol for 1.5 h. Filters were blocked by PBS with 0.2% tween 20/5% nonfat milk and probed using specific antibodies (Siriwardana et al. 2006). The proteins of interest were detected using the enhanced chemiluminescence (ECL) procedure. Uniformity of protein loading was measured by probing beta‐actin protein, after the membrane was stripped. Therefore, most *β*‐actin results are demonstrated in the figures.

Cell cycle measurements for assessment of apoptosis was assessed by flow cytometry, cells were stained with propidium iodide and analyzed by Fluorescence‐activated cell sorting (FACS) (Telford et al. 1992).

## Results

### DFO arrests cell division prior to and after the block by aphidicolin

**Figure 1.**
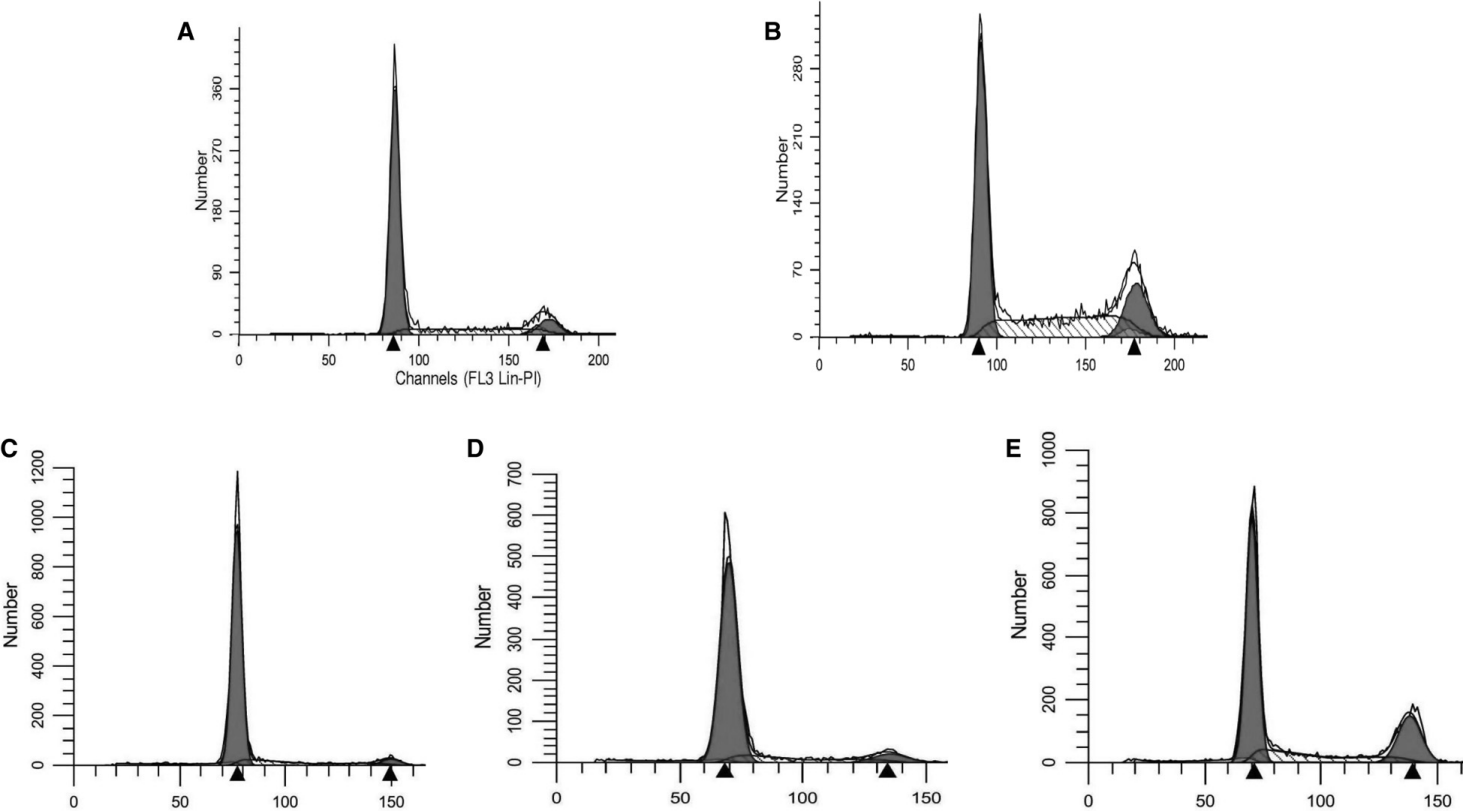
Cells were subcultured in 24‐well cell culture plates in RPMI with 10% FCS. FACS was conducted on samples of cells obtained at time of subculture (A) and 24 h later (B). Another set of cells were treated with DFO at time of subculture. One day later, these cells were released from DFO by replacing DFO medium with medium without DFO or other additions: control medium (CM). FACS was conducted of these cells at time of release (C), 6 h (D), and 24 h later (E).

Cultures of confluent SK‐N‐SH neuroblastoma cells were incubated in medium without serum (“serum starvation”) for 24 h (0 time) and cell cycle patterns were determined by flow cytometry at 0 time (Fig. 1A) and at various time points after subculture at low density in complete medium with or without various treatments. Cells untreated after subculture had entered S phase by 6 h and the majority (%) were in S and G2/M by 24 h (Fig. 1B). Cells subcultured in DFO for 1 day had a very narrow base at the G_1_ phase, an indication that the vast majority of the cells were at a diploid stage (Fig. 1C). However, 6 h after release from DFO, the base of the G_1_ or G_1_ phase widened indicating an increase in DNA content although most cells were measured as at G_1_/S or early S phase (Fig. 1D). At 24 h after release, 23% of the cells were in G2 and 19% were in S (Fig. 1E).

**Figure 2.**
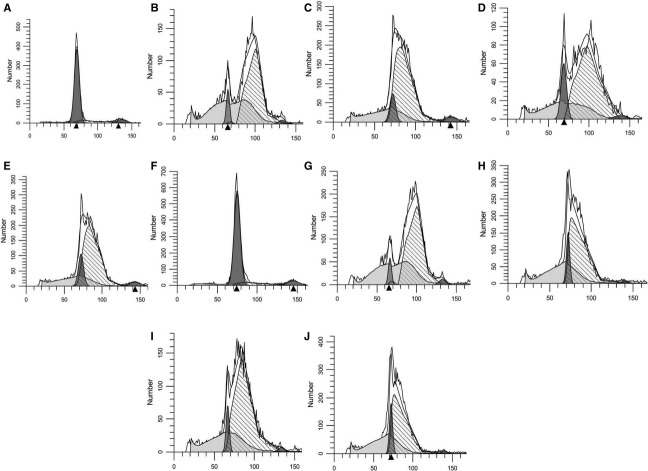
Cells were subcultured in 24‐well cell culture plates in RPMI with 10% FCS with aphidicolin or hydroxyurea. FACS was conducted on a set of cells that were in aphidicolin for 24 h (A). Another set of cells was released from aphidicolin by replacing with CM and were sampled 6 h later (B). A subset of cells that was in aphidicolin for 24 h was replaced with new medium containing DFO and incubated for another 24 h. After 24 h in DFO, these were released from DFO by replacing with CM and FACS was conducted on cells at time of release from DFO (C), and 6 h later (D). As a control, another set was replaced with new medium containing DFO and FACS was conducted 6 h later (E). FACS was conducted on a set of cells that were in hydroxyurea for 24 h (F). Another set of cells was released from hydroxyurea by replacing with CM and were sampled 6 h later (G). A subset of cells that was in hydroxyurea overnight was replaced with CM containing DFO and incubated for another 24 h. After 24 h in DFO, these were released from DFO by replacing with new medium and FACS was conducted on cells at time of release from DFO (H), and 6 h later (I). As a control, another set was replaced with new medium containing DFO and FACS was conducted 6 h later (J).

Cells that were treated with aphidicolin were in what was measured as G_1_ phase but with a wider base than DFO treatment (Fig. 2A compared to Fig. 1C). Six hours after release, 89% of the cells were in S phase (Fig. 2B). When treated with DFO after the removal of aphidicolin, the majority of the cells (85%) shifted but remained in S phase a day later without migrating to G2 phase indicating DFO caused a block after aphidicolin (Fig. 2C). However, after release from DFO, movement of cells toward G2 was visible with a shift within S phase (mean from 87 to 100) (Fig. 2D). Cells remaining in DFO did not shift (Fig. 2E). Cells blocked by hydroxyurea (Fig. 2F) and then released into CM for 6 h (Fig. 2G) showed a similar pattern to cells blocked by DFO after aphidicolin treatment and then released from the DFO (compare Fig. 2D with 2G).

Cells incubated in hydroxyurea overnight and replaced with new medium containing DFO showed a profile similar to that of cells that were treated with DFO after an overnight treatment with aphidicolin (compare Fig. 2C and H). Upon release from the DFO block, cells migrated toward G2 as indicated by the separation of the G_1_ peak from the S peak (Fig. 2I).

### Measurements of cyclin levels define the two DFO arrest points

**Figure 3.**
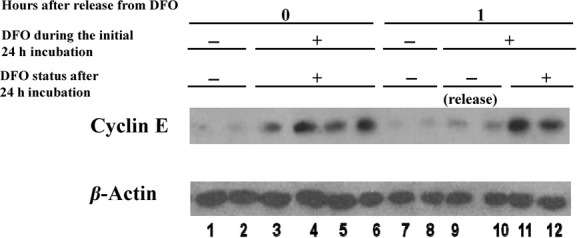
Cyclin E levels decrease after 1 h of release from the DFO block. Confluent neuroblastoma cells were serum starved for 24 h then split and plated into media, one set with DFO (lanes 3–6, 9–12) and one with no DFO (lanes 1–2, 7–8) and were incubated for 24 h. After 24 h, one set of cells that was in DFO was replaced with CM (release). Medium in the others were replaced with CM (continuously in medium without DFO). The medium in all plates was aspirated 1 h later and the cells were lysed using 200 *μ*L of hot SDS loading buffer. Forty microliter samples of the lysate were separated by 10% SDS‐PAGE and the proteins were transferred to PVDF membranes. This was first probed for cyclin E. Then the membrane was stripped and was probed for *β*‐actin. All treatments were conducted in duplicate.

Cyclin E and Cyclin A were measured. Synthesis of cyclin E peaks at early to mid‐G_1_ phase; cyclin A begins to be synthesized in late G_1_ and activates kinases necessary for S phase events (Lees et al. 1992; Sherr 1993; Kastan and Bartek 2004; MeSH Browser, 2011). Using western blot technique, protein E levels were not detectable in untreated cells at 24 h. However, after 24 h in DFO there was a marked increase in cyclin E protein compared to resting cells (Fig. 3). When these cells were released from DFO treatment cyclin E protein was greatly reduced within 1 h (Fig. 3) and not detectable after 2 h.

**Figure 4.**
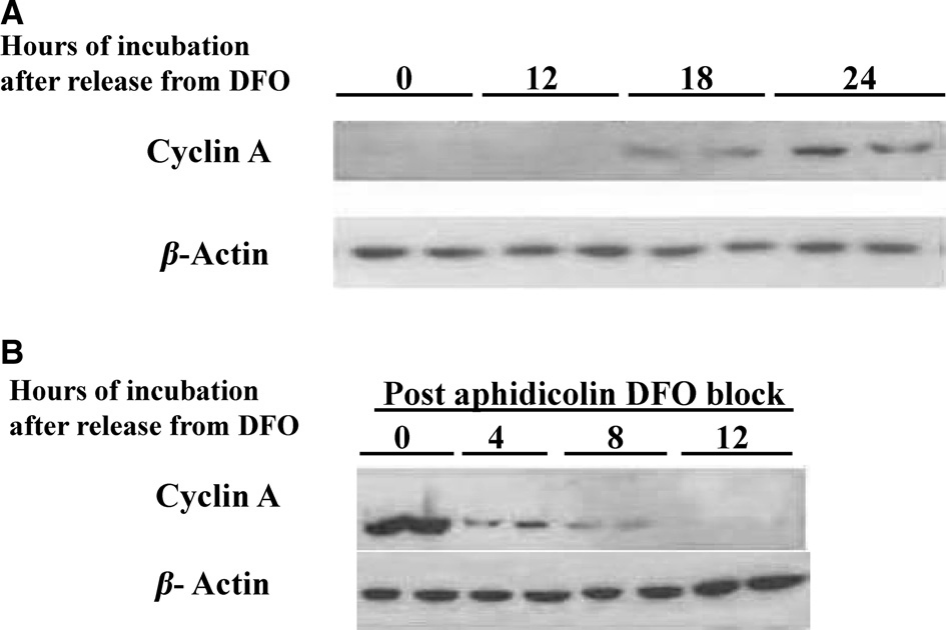
(A) Cyclin A protein steadily increases 12–24 h after release from DFO. Confluent neuroblastoma cells were serum starved for 1 day were and subcultured using RPMI/10% FCS with DFO. The plates were incubated for 20 h and then the medium was replaced with RPMI/10% FCS. The cells were harvested at the indicated times thereafter using 200 *μ*L of hot loading buffer. Forty microliter samples of the lysate were separated by 10% SDS‐PAGE and the proteins were transferred to PVDF membrane. This was first probed for cyclin A. Then, the membrane was stripped and was probed for *β*‐actin. All treatments were conducted in duplicate. (B) Ample cyclin A is present with the postaphidicolin DFO block. Serum starved neuroblastoma cells were subcultured in RPMI/10% FCS with aphidicolin and at 24 h medium was changed and contained DFO. Then, 24 h later medium was replaced with CM. Cell samples were then assayed by SDS‐PAGE as described in (A).

Cyclin A protein levels were barely detectable in serum deprived medium, cyclin A content with DFO treatment showed no change from starting cells (Fig. 4A). Cells subcultured into DFO and released a day later from DFO exhibited increased cyclin A protein from 12 to 24 h later (Fig. 4A). Cells blocked by DFO after aphidicolin treatment showed a relatively large amount of cyclin A at 24 h (Fig. 4B). The majority of this cyclin disappeared 4 h after release from DFO (Fig. 4B).

**Figure 5.**
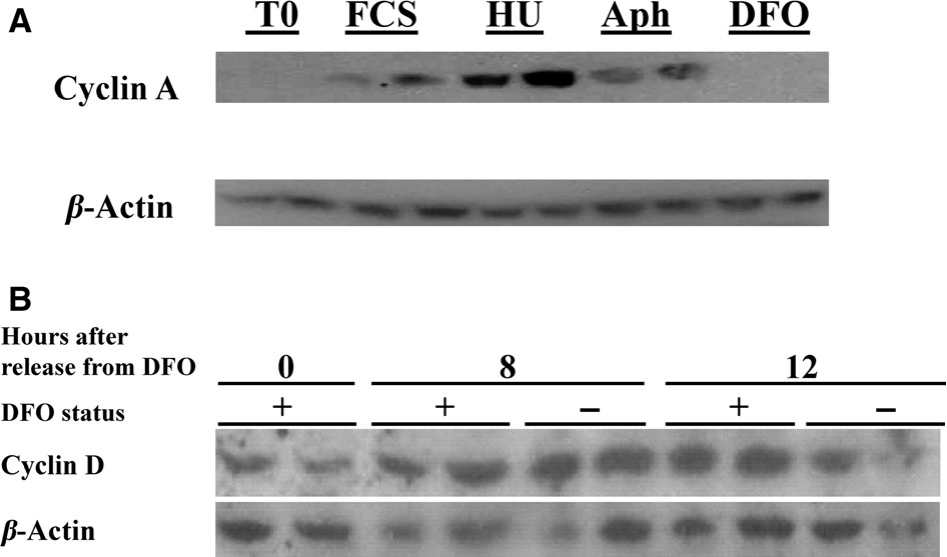
(A) Cyclin A is absent in DFO‐treated neuroblastoma cells but occurs in cells arrested with aphidicolin or hydroxyurea. Serum starved neuroblastoma cells for 24 h were subcultured in CM (FCS), or RPMI/10% FCS with hydroxyurea, aphidicolin or DFO. The dishes were incubated for 24 h and the cells were harvested in 0.5 mL of cold PBS, a 50 *μ*L portion of cells was used for FACS and the rest were centrifuged, supernatant removed and added with 200 *μ*L of hot SDS loading buffer. Forty microliter samples of the lysate were separated by 10% SDS‐PAGE and the proteins were transferred to PVDF membranes. This was probed for cyclin A. Then, the membrane was stripped and was probed for *β*‐actin. All treatments were conducted in duplicate. (B) Cyclin D1 protein levels are not affected by the DFO block. Confluent neuroblastoma cells were serum starved in medium with DFO. After 24 h, the media was replaced with RPMI/10%FCS with or without DFO. The cells were sampled at time of release from DFO, 8 and 12 h later. Gel electrophoresis was performed as detailed in (A).

Cyclin A content markedly increased in cells treated with either hydroxyurea or aphidicolin (Fig. 5A). As illustrated above, cyclin A is not measurable with DFO treatment under the same experimental conditions (Fig. 5A).

All of these data taken together indicate the earlier block seen with DFO as compared to the S phase block is before cyclin A synthesis and after the events that are needed for synthesis of cyclin E. We also examined earlier G_1_ events. Cyclin D1 protein content, the putative earliest G_1_ cyclin was present in resting cells and after incubation with DFO (Fig. 5B). Gel shifts indicated that at least some RB phosphorylation occurred in DFO‐treated cells (data not shown). This is in agreement with some cyclin D activity since cyclin D is (at least partially) responsible for early phosphorylation of RB (Sherr 1993; Huang et al. 2013).

### Further studies of SKNAS cells

**Figure 6.**
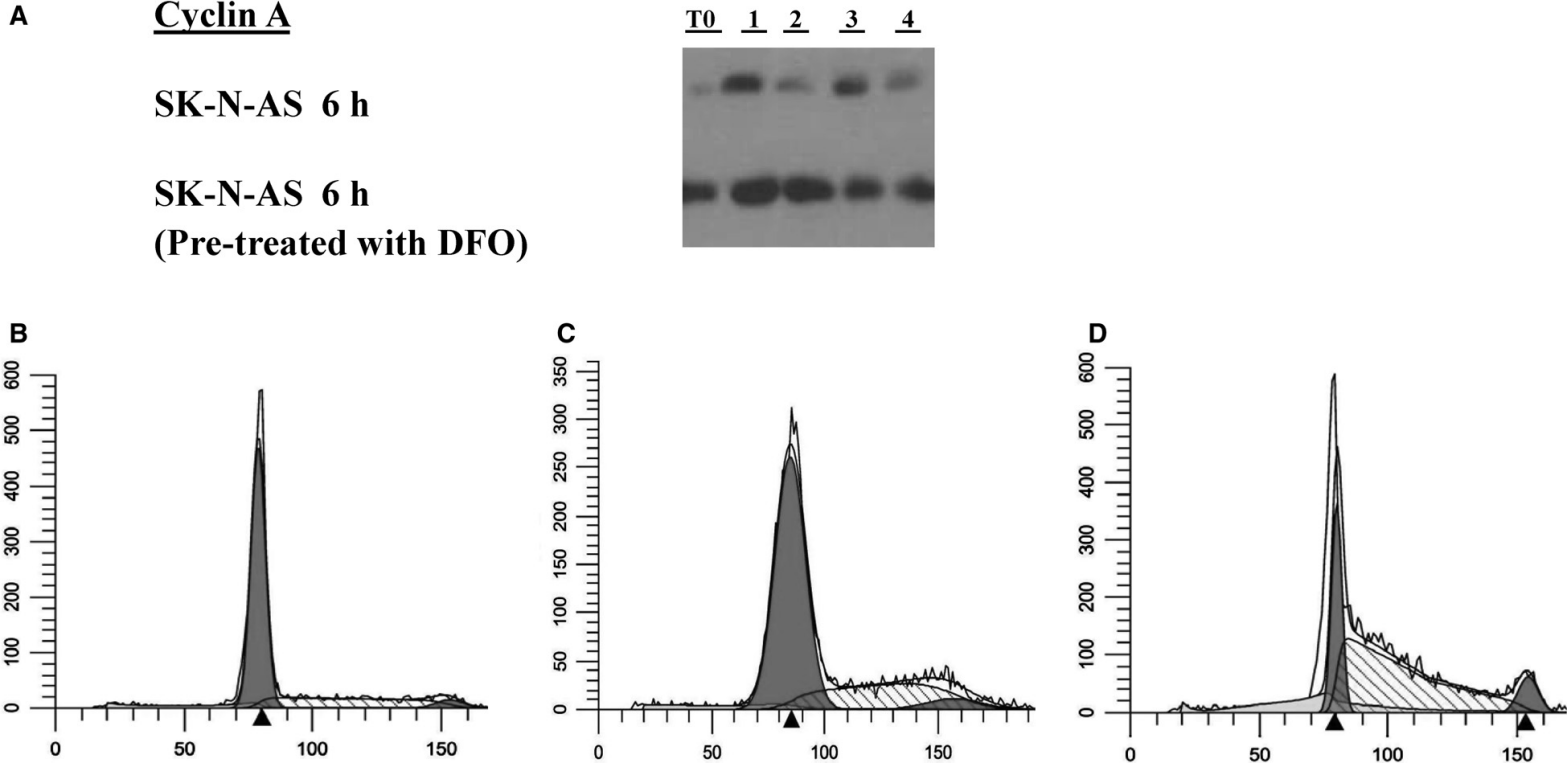
(A) Cyclin A measurements in SKNAS cells. The top gel shows Cyclin A measured in confluent cells in serum‐free medium for 1 day (T0). Duplicates of cells subcultured in DFO for 6 h (lanes 1 and 2) and cells subcultured in CM for 6 h (lanes 3 and 4). The lower gel shows confluent cells that were preincubated in DFO for 24 h (T0) and then duplicates of cells that were subcultured in DFO for 6 h (lanes 1 and 2) and cells that were subcultured in CM (lanes 3 and 4). (B) SKNAS cells with contact inhibition and no treatment analyzed for DNA content by FACS (methods detailed in Figs. 1, 2). (C) Cells subcultured in 100 *μ*mol/L DFO for 24 h. (D) Cells subcultured in 10 *μ*mol/L DFO for 24 h.

We observed that neuroblastoma cells SKNAS (AS) proliferated somewhat more rapidly than other cell lines, with serum starvation most of these cells are in G_1_ but with some measureable cyclin A protein expression (Fig. 6A). Cyclin A expression persists in these cells subcultured either in DFO or CM 6 h later, with similar results even if cells are preincubated in DFO (Fig. 6A). As noted above, the cells do not exhibit the G_1_ cell cycle arrest after subculture in DFO at 100 *μ*mol/L with early S phase arrest (Fig. 6B and C). AS also arrest in S at 10 *μ*mol/L DFO (Fig. 6D) doses similar to our prior studies that showed G_1_ arrest with SKNSH cells at 10 *μ*mol/L (Brodie et al. 1993). Higher DFO doses were needed for a glioblastoma cell line to show S phase arrest without G_1_ arrest suggesting at the time this difference was possibly due to increased resistance to DFO (Brodie et al. 1993). Results with SKNAS are similar to the results with hydroxyurea treatment of SKNSH with increased Cyclin A protein and G_1_/S phase arrest. Besides the higher cyclin A content in cells with DFO treatment, pCDK_2_ was not measurable in SH cells but was present in AS cells treated with DFO (data not shown).

### Inhibition of cellular proliferation of SKNSH at either the DFO G_1_ cell cycle block as well as the S phase cycle block are associated with a apoptosis

**Figure 7.**
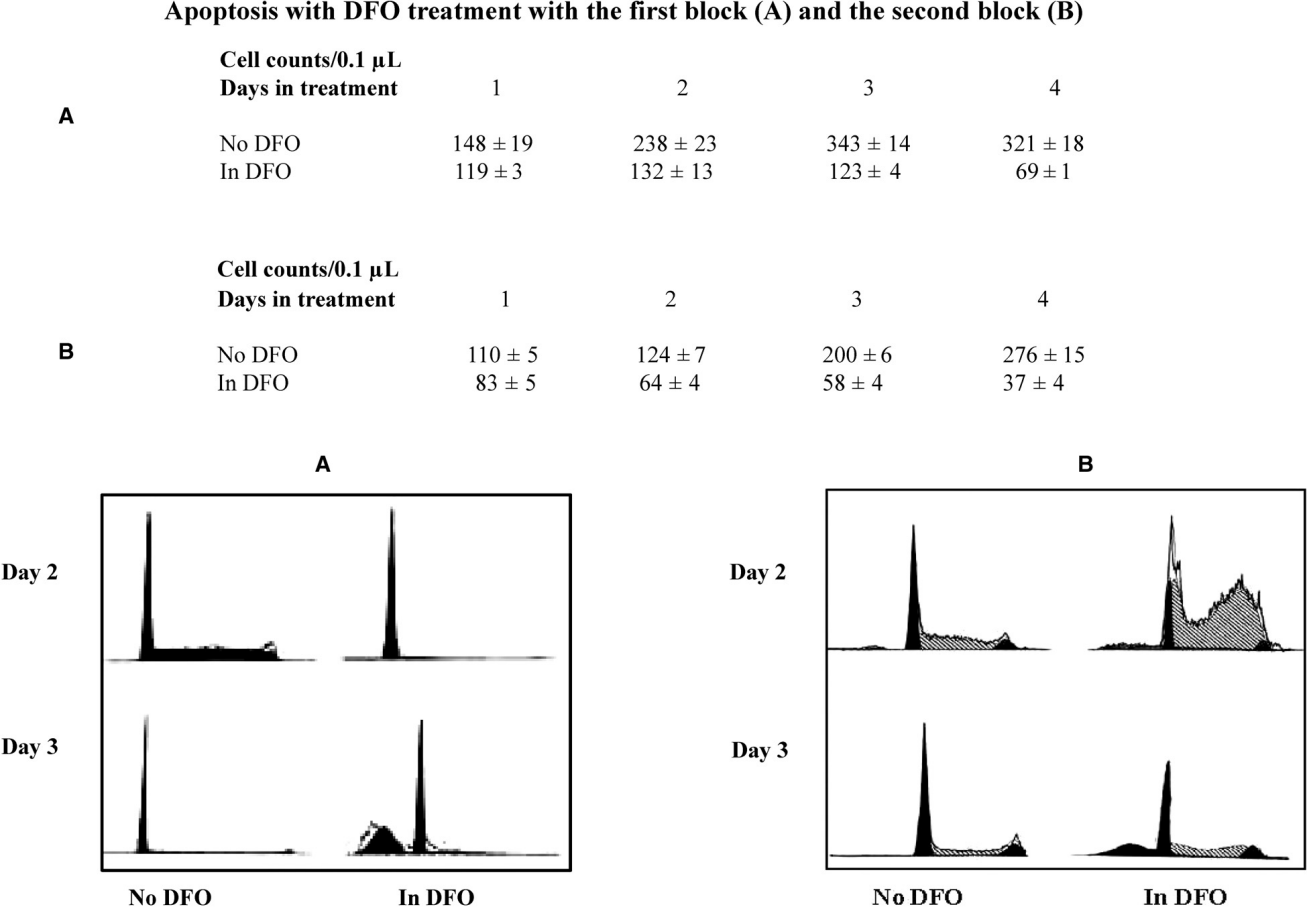
(A) The G_1_ block caused by DFO causes apoptosis in neuroblastoma. Confluent serum starved neuroblastoma cells (SKNSH) were subcultured in RPMI with or without DFO. The cells were harvested 2, 3, and 4 days later. For harvesting, the cells were washed with 0.5 mL of cold PBS once, scraped with a rubber policeman in 0.5 mL cold PBS. Twenty microliters of this was diluted to 100 *μ*L using PBS and a subsample of this was counted using the hemacytometer. Another 50 *μ*L was used to conduct cell FACS. Apoptotic cells are observed with DFO 3 days after treatment as a ‘hump’ to the left of the G1 cells (bottom row right). (B) Cells treated with DFO after aphidicolin treatment exhibit an S phase block and exhibit apoptosis a day later. Methods the same as shown in (A).

Untreated cells continue to grow through the fourth day after subculture. However, with DFO treatment, cell numbers remain static after 2 days and decline by 44% by the fourth day (Fig. 7A and B). Both the G_1_ and the S phase block associated with DFO treatment exhibited no apoptotic cells (by flow cytometry) for the first 2 days. However, on the third day 43% of cells treated with DFO that exhibit the G_1_ block were in apoptosis (Fig. 7A). DFO treatment resulting in S phase accumulation with the second DFO block showed that at 3 days 28% of cells were apoptotic (Fig. 7B).

## Discussion

Prior studies have documented that iron chelation by DFO and other chelators are associated with inhibition of cellular proliferation *in vitro* (Robbins and Pederson 1970; Lederman et al. 1984; Kontoghiorghes et al. 1986; Blatt and Stitely 1987; Helson and Helson 1992). Most of these earlier studies indicated that this effect was due in that inhibition of RR an enzyme required for DNA synthesis (see above), (Eriksson et al. 1984; Hoyes et al. 1992; Seguin et al. 2011; Zhang et al. 2011). We and others have previously shown that neuroblastoma cells are particularly sensitive to growth inhibition by DFO (Blatt et al. 1988; Brodie et al. 1993; Carosio et al. 2007).

Besides the well‐described S phase block associated with RR inhibition, a number of studies utilizing various cell lines including neuroblastoma have shown the growth arrest with iron chelation is associated with a block in G_1_ phase (Brodie et al. 1993; Nghia and Richardson 2002; Chaston et al. 2003; Carosio et al. 2007; Fu and Richardson 2007; Zhang et al. 2011). Under the experimental conditions, in this article, iron chelation of S KN‐SH cells exhibit cyclin D expression and probable activity as compared to other studies (Nurtjahja‐Tjendraputra et al. 2007) but cyclin E activity is inhibited. Our studies strongly indicate that this is the case since there is at least some RB phosphorylation with DFO treatment. Aphidicolin blocks DNA replication by inhibiting the activity of DNA polymerase, and therefore cells are considered arrested at G_1_/S (Sheaff et al. 1991), although some S phase protein changes may well be evident. In this study by treating SKN‐SH with DFO following aphidicolin treatment to define G_1_/S, the cells exhibit S phase arrest indicating RR inhibition with a similar DNA profile to the RR inhibitor hydroxyurea. This conclusion is supported by studies utilizing SKN‐AS, a rapidly growing cell line that that does not exhibit the G_1_ arrest point, but does show the S phase arrest with the indicated DFO treatment conditions. These conditions are similar to concentrations of DFO achieved *in vivo* when DFO is utilized for treatment of iron overload conditions (Hussain et al. 1977). Here, by separating the two arrest points we have devised a means to facilitate defining the unique events associated with each block.

The G_1_ arrest point is associated with accumulation of cyclin E protein, and the second arrest point in S phase exhibits increased cyclin A protein. Further studies of cell cycle regulatory proteins strongly indicate that the G_1_ arrest is after “start” but before G_1_/S (Lees et al. 1992; Sherr 1993; MeSH Browser, 2011).

Cyclin A production initially increases in cells during late G_1_ phase (MeSH Browser, 2011). Our observations suggest that cyclin A is first detected in neuroblastoma cells about 12–18 h after release from serum starvation and/or DFO treatment and therefore before G_1_/S. Although it may be suggested that iron chelation can cause a direct effect on cyclin A synthesis, the most obvious explanation for the iron chelation effect at the G_1_ arrest point causes impaired activity of cyclin E by the continued presence of a direct inhibitor of cyclin E activity or changes in substrate recognition causing inhibition of phosphorylation of CDK2 by the CDK2 cyclin E complex (Fischer 2001; Ye et al. 2003). Alternatively, a number of specific inhibitors have been described that directly or indirectly interfere with CDK2 phosphorylation including p16, p21, and p27 (Sherr 1993; Hengst and Reed 1996; Hengst et al. 1998; Fischer 2001; Fu and Richardson 2007). By separating the changes that occur with the G_1_ arrest point compared to the S phase arrest point, the contribution of any or all of these possibilities can be better defined.

Although apoptosis has been described as an effect of iron chelation (Greene et al. 2002; Yu et al. 2012), we demonstrate that definitive separation of both blocks caused by DFO treatment of neuroblastoma cells is associated with apoptosis. This effect may have clinical importance for neuroblastoma or other malignancies since cells affected at two points in the cell cycle may allow for increased efficacy clinically especially with the use of combination therapy with iron chelation. Any *in vivo* study should document interference with iron metabolism (Krokan et al. 1981; Lui et al. 2013). The availability of new oral chelators would make these combination treatments more practical and effective (Chaston et al. 2003; Choi et al. 2012; Yu et al. 2012; Ford et al. 2013; Lui et al. 2013).

## Acknowledgments

We acknowledge the technical assistance of Rhoda Schleicher in laboratory studies. We acknowledge Theresa M. Martinez for her assistance with manuscript preparation.

## Conflict of Interest

None declared.
